# 4274 Thirteen Years of Pipeline Programming at the University of Rochester’s Clinical & Translational Science Institute to Train Physician-Scientists

**DOI:** 10.1017/cts.2020.228

**Published:** 2020-07-29

**Authors:** Alaina Maiorano, Edwin van Wijngaarden, Alfred Vitale, Timothy De Ver Dye, Robert Gross, Kerry O’Banion

**Affiliations:** 1 University of Rochester Medical Center

## Abstract

OBJECTIVES/GOALS: Physician-scientists play a vital role in biomedical research but this chosen career path has many challenges, such as long training periods and funding. The University of Rochester (UR) CTSI pipeline programs address this by enabling medical trainees to partake in enriched research experiences. METHODS/STUDY POPULATION: The UR CTSI TL1 is a training grant from the National Center for Advancing Translational Science (NCATS), which funds predoctoral trainees. The TL1-funded physician-scientist pipeline includes the Academic Research Track (ART) year-out program and the Medical Science Training Program (MSTP). We describe the characteristics and training outcomes of TL1-funded trainees. We also obtained testimonials of current and former trainees regarding their career component decision-making, and their perception of programs, in order to identify how best to address the challenges of the physician-scientist workforce, and to facilitate the transition between the clinic and bench. RESULTS/ANTICIPATED RESULTS: From 2006-2019, the UR CTSI has had 56 ART trainees and 17 MSTP trainees complete training; six trainees have transitioned into the MSTP after completing the ART program. As of 2019, 63 of 67 graduated trainees (94%) have continued their engagement in CTS after graduation. Importantly, our programs have facilitated the careers of 31 women (39.7%) and 12 under-represented minorities (15.4%). We will present a breadth of qualitative data to inform which parts of the TL1-related programs have been successful, and which parts could use programmatic improvement to aid the transition into the physician-scientist workforce. DISCUSSION/SIGNIFICANCE OF IMPACT: Physician-scientist training barriers in the US have resulted in a shortage of these professionals in the clinical and translation workforce. Our data show the UR CTSI has been successful in addressing several of these challenges via the TL1-funded ART, MSTP, and ART/MSTP dual program pipeline.

